# Reply to Rhazzar et al. The Impact of Author Name Ambiguity on Bibliometric Validity. Comment on “Ejaz et al. Bibliometric Analysis of Publications on the Omicron Variant from 2020 to 2022 in the Scopus Database Using R and VOSviewer. *Int. J. Environ. Res. Public Health* 2022, *19*, 12407”

**DOI:** 10.3390/ijerph23050582

**Published:** 2026-04-30

**Authors:** Hasan Ejaz, Hafiz Muhammad Zeeshan, Fahad Ahmad, Syed Nasir Abbas Bukhari, Naeem Anwar, Awadh Alanazi, Ashina Sadiq, Kashaf Junaid, Muhammad Atif, Khalid Omer Abdalla Abosalif, Abid Iqbal, Manhal Ahmed Hamza, Sonia Younas

**Affiliations:** 1 Department of Clinical Laboratory Sciences, College of Applied Medical Sciences, Jouf University, Sakaka 72388, Saudi Arabia; 2 Department of Computer Sciences, National College of Business Administration and Economics, Lahore 54700, Pakistan; 3 Department of Basic Sciences, Deanship of Common First Year, Jouf University, Sakaka 72388, Saudi Arabia; 4 Department of Pharmaceutical Chemistry, College of Pharmacy, Jouf University, Sakaka 72388, Saudi Arabia; 5 Allied Health Department, College of Health and Sport Sciences, University of Bahrain, Zallaq 32038, Bahrain; 6 Department of Computer Science, Lahore Leads University, Lahore 54000, Pakistan; 7 School of Biological and Behavioural Sciences, Queen Mary University of London, London E1 4NS, UK; 8 Department of Library, Prince Sultan University, Riyadh 11586, Saudi Arabia; 9 Department of Medical Microbiology, Faculty of Medical Laboratory Sciences, Omdurman Islamic University, Omdurman 14415, Sudan; 10 HKU-Pasteur Research Pole, School of Public Health, LKS Faculty of Medicine, The University of Hong Kong, Hong Kong, China

We sincerely thank the authors of the comment for their interest in our study and for highlighting the issue of author name ambiguity in bibliometric research [1]. We appreciate the opportunity to clarify the methodological intent, scope, and interpretation of our findings, considering the points raised.

## 1. Response to the Comment on Author Name Ambiguity

The comment correctly points out that author name ambiguity is a known limitation in bibliometric analyses, particularly when dealing with common surnames and initials. We fully acknowledge that bibliographic databases such as Scopus may aggregate publications from different individuals under identical author name strings (e.g., “Wang Y.” or “Zhang Y.”). This issue has been widely documented in the bibliometric literature and represents a structural limitation of large-scale database-driven analyses rather than a flaw specific to our study.

As clearly stated in the Materials and Methods section, our work employed Scopus-indexed metadata analysed using Bibliometrix/Biblioshiny (R) and VOSviewer, following established and widely accepted bibliometric practices. The objective of the study was to provide a macro-level quantitative overview of Omicron variant research, focusing on publication growth, thematic evolution, country-level contributions, institutional productivity, and collaboration patterns, rather than on individual-level author attribution or academic evaluation.

## 2. On the Interpretation of “Most Productive Authors”

The identification of “most relevant” or “most productive” authors in our results reflects author name entities as indexed in Scopus, not verified individual researcher identities. This distinction is inherent to bibliometric analyses that operate at scale and rely on automated metadata processing. Importantly, our manuscript does not claim that these author labels correspond to single, uniquely identifiable individuals, nor does it use these results to evaluate researcher performance, expertise, or academic impact at the personal level.

Moreover, the author-level results represent only one descriptive component of a broader analysis. The core contributions of the study lie in:

- Mapping global research output on the Omicron variant over time;
- Identifying leading countries and institutions;
- Visualising collaboration networks;
- Analysing keyword co-occurrence, thematic clusters, and conceptual structures.

These analyses are not affected by author name ambiguity, as they rely on aggregate patterns rather than individual author identification.

## 3. On Methodological Transparency and Study Limitations in the Context of Bibliometric Practice

We agree that explicitly discussing author name ambiguity can enhance methodological transparency. At the same time, it is important to note that many bibliometric analyses published since 2020, including studies conducted using Scopus- or Web of Science-based datasets and tools such as VOSviewer, primarily report limitations related to database coverage, indexing policies, language restrictions, and inclusion criteria, without explicitly identifying author name disambiguation as a separate limitation.

For example, Yu et al. (2020) conducted a bibliometric analysis of COVID-19 research using VOSviewer and discussed limitations related to database indexing and the exclusion of preprints, without explicitly addressing author name ambiguity [2]. Similarly, Yıldızhan and Çelik (2024), in their bibliometric study of TRPM2 research, included a strengths and limitations section focused on database scope and methodological constraints, but did not explicitly flag author name disambiguation [3]. More recently, Arokiasamy et al. (2024) conducted a Web of Science–based bibliometric analysis of sustainable employability research (2014–2024), focusing on publication trends, thematic hotspots, collaboration patterns, and methodological characteristics, without explicitly identifying author name ambiguity as a standalone limitation [4].

These examples reflect a common and accepted methodological convention in bibliometric research, particularly when the emphasis is on macro-level trends and structures rather than individual-level attribution. In line with this practice, our study adopted standard bibliometric workflows and tools and did not aim to evaluate individual researcher identities or performance. Nevertheless, we acknowledge that future bibliometric studies, including potential extensions of our own work, may benefit from:

- Explicit mention of the author’s name ambiguity as a known limitation;
- Use of ORCID identifiers where consistently available;
- Complementary analyses at the institutional or country levels to mitigate individual-level ambiguity.

## 4. Validity and Contribution of the Study

We respectfully emphasise that the concern raised in the Comment does not invalidate the study’s findings or conclusions. The paper provides a reproducible, transparent, and quantitative snapshot of Omicron-related research during a critical period (2020–2022), based on 1917 publications indexed in Scopus. The insights into publication trends, collaboration patterns, and thematic evolution remain robust and informative for researchers, policymakers, and public health stakeholders.

## 5. On the Role of Databases and Bibliometric Tools in Addressing Author Name Ambiguity

We further note that different bibliographic repositories and analytical tools offer complementary mechanisms to mitigate author name ambiguity. Major databases such as Scopus and Web of Science increasingly support author profiling through unique author identifiers, affiliation histories, and citation linkages. The integration of ORCID identifiers, when consistently adopted, provides a reliable mechanism for distinguishing individual researchers across publications.

From an analytical perspective, tools such as Bibliometrix/Biblioshiny (R), VOSviewer, and CiteSpace support aggregation and network-based analyses that prioritise institutional, country-level, and thematic patterns, thereby reducing reliance on individual author attribution. Advanced workflows combining multiple databases or hybrid algorithmic and manual validation approaches may further improve disambiguation, although such methods often require substantial effort and are not always feasible for large-scale, time-bound studies.

## 6. Concluding Remarks

We appreciate the Comment for drawing attention to an important methodological consideration in bibliometric research. We believe this exchange contributes constructively to scholarly discussion on bibliometric best practices. While acknowledging the inherent limitation of author name ambiguity, we maintain that the scope, methodology, and conclusions of our study remain valid, appropriate, and aligned with established bibliometric standards.
